# UC.183, UC.110, and UC.84 Ultra-Conserved RNAs Are Mutually Exclusive with miR-221 and Are Engaged in the Cell Cycle Circuitry in Breast Cancer Cell Lines

**DOI:** 10.3390/genes12121978

**Published:** 2021-12-13

**Authors:** Fabio Corrà, Francesca Crudele, Federica Baldassari, Nicoletta Bianchi, Marco Galasso, Linda Minotti, Chiara Agnoletto, Gianpiero Di Leva, Federica Brugnoli, Eva Reali, Valeria Bertagnolo, Andrea Vecchione, Stefano Volinia

**Affiliations:** 1 Laboratorio per le Tecnologie delle Terapie Avanzate (LTTA), Department of Translational Medicine, University of Ferrara, Via Fossato di Mortara 70, 44121 Ferrara, Italy; corra.fabio@gmail.com (F.C.); crdfnc@unife.it (F.C.); federica.baldassari@unife.it (F.B.); nicoletta.bianchi@unife.it (N.B.); galasso.marco84@gmail.com (M.G.); felinda086@gmail.com (L.M.); federica.brugnoli@unife.it (F.B.); valeria.bertagnolo@unife.it (V.B.); 2 Advanced Translational Research Laboratory, Veneto Institute of Oncology IOV-IRCCS, 35127 Padua, Italy; chiara.agnoletto@iov.veneto.it; 3 School of Pharmacy and Bioengineering, Guy Hilton Research Centre, Keele University, Stoke-on-Trent ST4 7QB, UK; g.dileva@keele.ac.uk; 4 Department of Biotechnology and Biosciences, University of Milano-Bicocca, 20126 Milan, Italy; eva.reali@grupposandonato.it; 5 Department of Medical Surgical Science and Translational Medicine-c/o Azienda Ospedaliera Sant’Andrea, Via di Grottarossa 1035, 00189 Rome, Italy; andrea.vecchione@uniroma1.it

**Keywords:** UCR, MIR221, breast cancer, cell cycle, capivasertib, alpelisib, voxtalisib

## Abstract

In the human genome, there are about 600 ultra-conserved regions (UCRs), long DNA sequences extremely conserved in vertebrates. We performed a large-scale study to quantify transcribed UCR (T-UCR) and miRNA levels in over 6000 cancer and normal tissue samples to find possible correlation between these kinds of regulatory molecules. Our analysis evidenced several non-coding RNAs showing negative co-regulation with miRNAs; among them, we focused on miR-221 to investigate any relationship with its pivotal role in the cell cycle. We have chosen breast cancer as model, using two cell lines with different phenotypes to carry out in vitro treatments with siRNAs against T-UCRs. Our results demonstrate that the expression of uc.183, uc.110, and uc.84 T-UCRs is mutually exclusive with miR-221 and is engaged in the regulation of *CDKN1B* expression. In addition, tests with a set of anticancer drugs, including BYL719, AZD5363, AZD8055, AZD7762, and XL765, revealed the modulation of specific T-UCRs without alteration of miR-221 levels.

## 1. Introduction

Non-coding RNAs (ncRNAs) represent a large portion of the human genome which are not translated into proteins mediating transcriptional gene modulation [1]. Many non-coding RNAs contribute to the alteration of biological functions in normal cells, leading to progression and malignant phenotype in cancer [2]. Among them, the class of Ultra-conserved regions (UCRs) are DNA elements of more than 200 base pairs long, without insertion or deletion and extremely conserved in the orthologous loci of vertebrates, in particular human [3], mouse, and rat genomes [4], but Single Nucleotide Polymorphisms (SNPs) in UCRs are related to cancer susceptibility [5]. Their expression is altered in leukemia [6], liver cancer [7], glioma [8], and neuroblastoma [9], which might be modulated either by promoter hyper-methylation or by interactions with microRNAs (miRNAs) [10]. The Transcribed-UCRs (T-UCRs) are a class of non-coding RNAs and are involved in gene expression regulation transcription [11] and splicing [12] during development processes. There is a considerable overlap between T-UCRs and long non-coding RNAs (lncRNAs) [13,14]. The biological functions of lncRNAs are ascribable to control and regulation of cell cycle cell, metabolism, immune response [15], differentiation [16], and transcription/translation [17], but they can also regulate cancer onset, progression, or survival of patients [18,19,20,21]. One T-UCR seems to regulate apoptosis [22]; however, in the literature, there are few reports about the influence of other T-UCRs in this cellular program.

MiRNAs, a subgroup of non-coding RNAs, can enable oncogenes or inactivate onco-suppressors in solid cancers [23]. Consistently, Pineau et al. demonstrated that miR-221/miR-222, the most upregulated miRNA in hepatocarcinoma, dysregulated cell growth by targeting the CDK inhibitor p27 [24]. Furthermore, the same miR-221/miR-222 have a strong effect on cell cycle with the promotion of G1/S transition and contribute to aggressiveness of breast cancer (BC) [25].

In this study, we investigated the genome-wide expression of all UCRs, analyzing the T-UCRs levels in a very large dataset of human normal and cancer samples. Thus, we identified strong T-UCRs candidates for cell cycle regulation using the expression of miR-221 as a ‘bait’. Then, we employed siRNAs against T-UCRs to evaluate their impact on cell cycle regulation, focusing on their interactions with miR-221 and on some other key effectors of cell cycle. With this aim, we further investigated the T-UCRs’ expression upon treatments of BC cell lines using anticancer drugs, which led to the identification of an alternative modulation of miR-221 and T-UCRs.

## 2. Materials and Methods

### 2.1. Data Mining of miRNA and T-UCRs Expression Profiles

We studied the expression of T-UCRs and miRNAs in 6604 samples, derived from cancer and control tissues, using the Ohio State University Comprehensive Cancer Center (OSUCCC) custom microarray [8,23]. Two sub-cohorts of identical size (each one consisting of 3302 samples), a test and a validation dataset, were generated by random selection. The interquartile range (IQR) was used as a threshold to remove T-UCRs and miRNAs with low variability. Linear correlation (Pearson) and mutual information content (MIC) [26] were used to assay co-regulation of miR-221 expression with T-UCRs and other miRNAs, and thus detect candidate alternatives/antagonists. Custom made scripts were coded using Python and R.

### 2.2. Cultures, Cell Cycle Synchronization, Silencing, and Drug Treatments

We used two breast cancer derived cell lines, MCF-7 and MDA-MB-231. MCF7 has a luminal A profile (ER+, PR+, HER2−) and wild type TP53, with a low proliferation rate and a low capacity of invasion. MDA-MB-231 belongs to the basal mesenchymal-like triple negative subtype presenting mutated TP53 with high proliferative and invasiveness potential [27,28]. Cells were cultured in Dulbecco’s modified Eagle’s medium DMEM (GE-Healthcare) supplemented with 10% FBS, 2 mM L-Glutamine and 50 U/mL Penicillin and 50 μg/mL Streptomycin (Sigma-Aldrich, Milan, Italy).

The DNA content was evaluated to determine the percentage of cells in the different cell cycle stages. Fluorescence emitted from the propidium iodide–DNA complexes was quantified by the MUSE analyzer and the cell cycle kit (Luminex Corporation, Austin, TX, USA).

RNA interference experiments were carried out targeting selected T-UCRs, as reported in Table S1. The cells were transfected with 75 nM of a specific siRNA directed against the T-UCRs elements or against hsa-miR-221-3p (5′-AGCUACAUUGUCUGCUGGGUUUC-3′) [29]. Anti-miR-221 (5′-GAAACCCAGCAGACAAUGUAGCU-3′) [29] and a random pool of siRNAs were respectively used as positive or negative control [30] (Fidelity Systems Inc., Gaithersburg, MA, USA). Approximately 100,000 cells/well were cultured in 6-well plates with complete medium 10% FBS and after 16 h the medium was replaced with 0.1% FBS-containing medium. Transfection with siRNA molecules was then performed using the siPORT transfection agent (Life Technologies, Monza, Italy) according to the manufacturer’s instructions.

For cell cycle synchronization in G0/G1 phase, we used two different setups: double thymidine block [31] or serum starvation for 48 h [32]. For the double thymidine block, cells were treated 18 h with 2 mM thymidine (Sigma-Aldrich, Milan, Italy), then washed twice with complete medium and incubated for additional 8 h (to release them from the first thymidine block). Subsequently, cells were treated again with 2 mM thymidine for 15 h before the second release. Finally, the cells were collected at 2 different times, i.e., at the end of the block (T0, release) (cell arrested in G0/G1 phase) and 8 h later (T8). For serum starvation, cells were maintained in 0.1% FBS medium for 48 h and harvested 8 h after replacement with complete medium. The BC cell lines were treated using 14 different anticancer drugs (Chemietek, Indianapolis, IN, USA), selected to target the major dysregulated pathways in BC and used at half maximal inhibitory concentration (IC50), as reported by Baldassari et al. [33]. After 24 h of exposure to drugs, total RNA was collected using Trizol^TM^ (Invitrogen, Monza, Italy).

### 2.3. Quantitative RT-PCRs

To analyze RNA expression, Reverse Transcription (RT) was performed using 400 ng of total RNA and oligo-dT plus random primers with the Superscript II enzyme (Invitrogen, Monza, Italy). Quantitative PCR (qPCR) was carried out using the power SYBR Green PCR master mix (Applied Biosystems, Foster City, CA, USA) with the primer pairs listed in Table S2. Reactions were first incubated at 50 °C for 2 min and then at 95 °C for 2 min, followed by 40 cycles, each at 95 °C for 15 s and at 60 °C for 1 min, on a Bio-Rad CFX thermal cycler. Each sample was analyzed in duplicate. β-actin was used as the endogenous reference gene. The RNA levels were assessed as relative expression values measured using ΔΔCq (Bio-Rad CFX Manager Software, version 3.1). The log2 fold changes (2^−ΔΔCq^) were calculated and compared to control samples. MiR-221 RT-qPCRs were performed following the protocol described by Wang et al. [34].

### 2.4. Statistical Analysis

The qPCR data were normalized using mock transfections and analyzed applying two-tailed unpaired Student’s *t*-test as calculated by Bio-Rad CFX Manager Software (version 3.1), with significant adjusted *p*-values < 0.05. As control for multiple testing in the drug treatments, we used the Benjamini–Hochberg correction (FDR < 0.05). Cell cycle results were obtained from at least three independent experiments and analyzed using the Mann–Whitney U test.

## 3. Results and Discussion

### 3.1. Identification of T-UCRs Alternatively Expressed with miR-221

**Figure 1:**
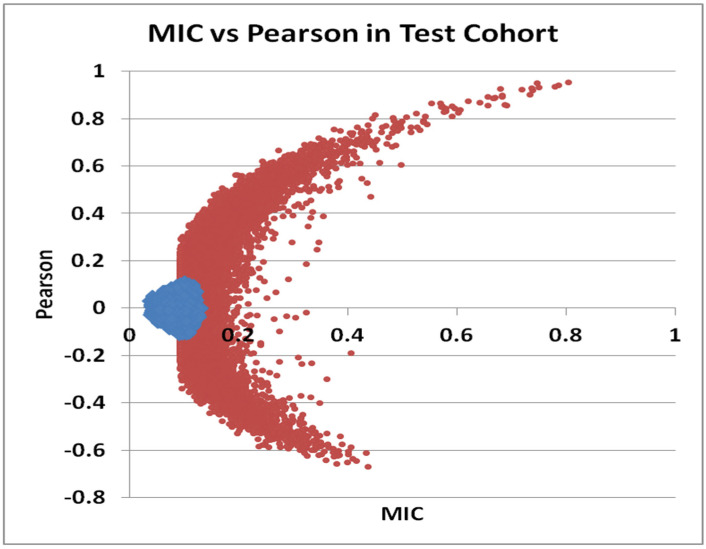
Scatter plot of Maximal Information Coefficient and Pearson correlation of ncRNAs in the Test cohort (*n* = 3302). The values for 40,486 pairs of ncRNAs (T-UCRs and miRs) are reported in red. In blue are also plotted the values for the simulation.

We performed a genome-wide study of T-UCRs expression with the aim to identify novel ncRNAs involved in the human cell cycle. We used approaches from information theory and statistics, respectively Maximal Information Coefficient (MIC) [35] and Pearson correlation, to reveal any significant co-regulation between the expression of T-UCRs and miRNAs. The two data mining approaches we used were as distant as possible, although it has been previously reported that there still is a strong correlation between Pearson r and MIC [35]. We took advantage of a large dataset of T-UCRs and miRNA expression profiles, derived from 6604 human samples of cell lines, cancers and normal tissues [2], and randomly divided in two sub-sets representing a Test and a Validation cohort, each one containing 3302 samples. IQR was used to discard the ncRNAs with lowest variation. Finally, we retained the expression measures for 860 genome elements, either T-UCRs or miRNAs, expressed above background in at least 255 samples. We then proceeded to identify the strongest, positive, or negative, co-regulations in the Test cohort. A permutation analysis was used to simulate the noise in the procedure and generate confidence intervals. Depending on the role of the T-UCR, or its position in the transcriptional cascade, we would detect either a positive or a negative correlation score with miR-221. The scatter plot of all Pearson r and MIC score obtained in the test cohort (red points) and in the simulation (blue points) is shown in Figure 1.

The same procedure was performed in the Validation cohort, essentially confirming the results of the Test cohort. Of note these measures provided a profile of the cellular steady-state, as basically no time courses were used but only tissues and cell cultures.

As expected from a structured genome geared towards maintaining homeostasis, most of the real-world interactions (red dots) between miRNA: miRNAs, miRNAs:T-UCR, and T-UCR:T-UCR are located away from the noise (blue dots). Additionally and reassuringly, miR-222 (co-localized with miR-221 at Xp11.3) was the ncRNA with the maximum positive r and MIC in conjunction with miR-221. The miR-221/miR-222 relation was plotted as a red point at the top and right-hand quadrant of Figure 1, together with other cell cycle and miR-221 co-regulated ncRNAs. Conversely, the values for alternative ncRNA associations are graphed as red points in the right-hand side and lower quadrant of the distribution. In the following step, we focused on ncRNAs which could act as cellular alternatives, or even antagonists, to miR-221. Thus, we selected the T-UCRs/miR-221 pairs with MIC larger than 0.2 and Pearson r lower than −0.4, as listed in Table 1.

The most relevant T-UCRs, candidate as miR-221 alternatives/antagonists, are listed in Table 2.

Finally, we used the miRDB [46] online tool to verify whether any of these T-UCR sequences could bear predicted targeting sites for miR-221, or miR-222. We further extended this investigation applying the RNA22 [47] and PITA [48] algorithms, but no targets for miR-221 or miR-222 were detected, suggesting that the microRNA and the T-UCRs could be indirectly linked, perhaps through an indirect transcriptional control.

### 3.2. Analysis of T-UCRs Involvement in the Cell Cycle of BC Cells

We performed in vitro experiments to evaluate the possible role of the T-UCRs associated with negative co-regulation of miR-221, in relation to cell cycle and to quantify their levels in different cell cycle phases. We designed specific siRNA molecules against T-UCRs, one pair for each strand, as reported in Table 2, and assayed their silencing potential on MCF-7 and MDA-MB-231 cells. Since miR-221 strongly affects cell cycle promoting G1/S transition, we investigated whether these siRNAs showed comparable activity. We performed a primary screen of these thirteen candidate T-UCRs using siRNA pools (Figures S1–S3), and chose uc.183, uc.110, uc.96, and uc.84 (Supplementary Materials, Figures S4–S7) for further validation. Their expression was quantified in unsynchronized MCF-7 and MDA-MB-231 cells (basal levels reported in Figure S8), as well as upon double thymidine block or serum starvation (Table S3). The results confirmed that miR-221 transcription was abundant in MDA-MB-231, as previously reported [25]. Consistently, the levels of both pre-miR-221 and miR-221 were increased at T8 (8 h from block release) in synchronized MCF-7 and MDA-MB-231 cells, while the levels of uc.183, uc.110, and uc.96 were decreased when cell cycle was blocked using double thymidine or serum starvation. Such pattern was thus in agreement with the inverse correlation between these T-UCRs and miR-221 expression detected in the Test cohort.

**Figure 2:**
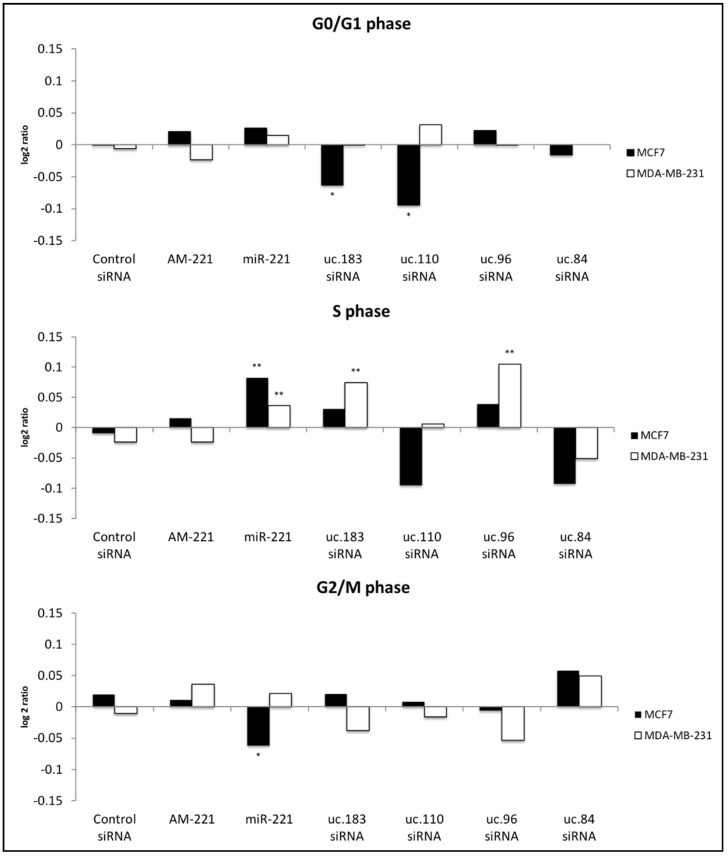
RNA interference of uc.84, uc.96, uc.110, and uc.183 on cell cycle in BC synchronized cell lines. Cell cycle was analyzed after transfections with siRNAs against the selected T-UCR, with miR-221 or anti-miR-221 (AM-221). Quantification was plotted as log2 ratio (median). Statistical significance was calculated, and the result compared to control random siRNA by 2-tailed Mann–Whitney test. *p*-values < 0.05 (*), *p*-values < 0.01 (**).

Focusing our attention on the relationship between T-UCRs and miR-221, we carried out experiments of silencing in each synchronized cell line, and assaying cell cycle phases using the MUSE cell analyzer. If a siRNA against T-UCRs was effective, it would show an effect similar to that observed with miR-221. As described in Figure 2, uc.183 and uc.96 both revealed such a miR-221-like activity, leading to significant increase of MDA-MB-231 cells in the S phase.

Analogously, the same trend of uc.183 and uc.96 was detected in MCF-7 cells; however, the data were not significant, maybe depending on the higher basal levels in this kind of cells as occurred in the case of treatment with anti-miR-221 (see Figure 2, phase S). The effects on cell cycle by uc.183 and uc.110 siRNAs, and by miR-221 transfection were confirmed when considering all data independently from the cell line (*p* < 0.05). We also provide a representation of the mean fold change of cell cycle data ± SEM in Figure S9.

**Figure 3:**
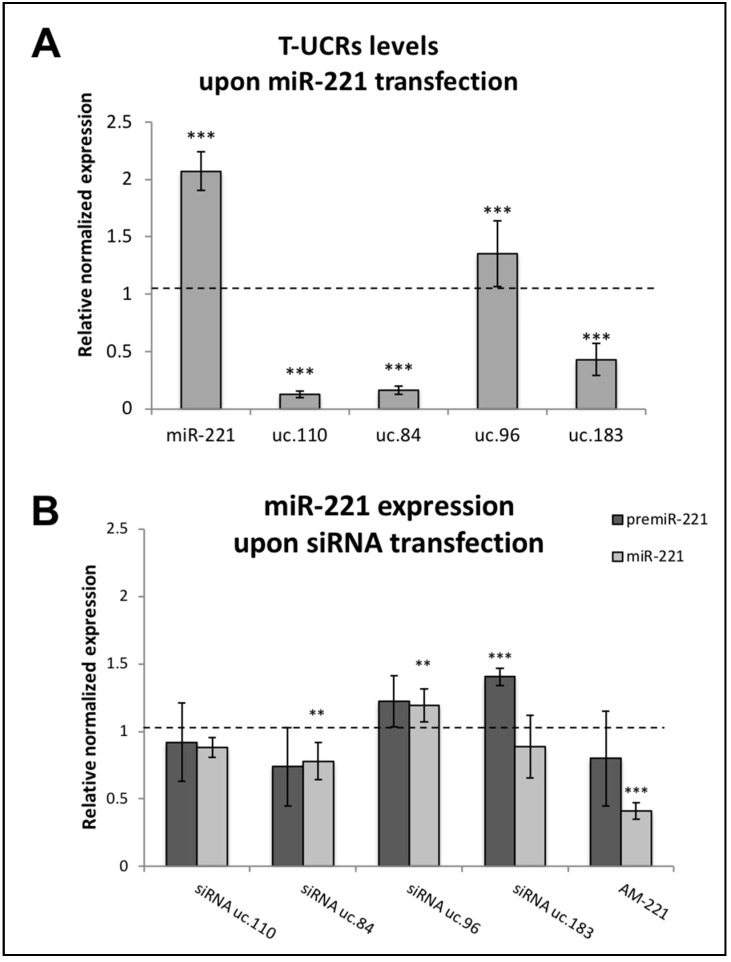
Effects of downregulation of T-UCRs or miR-221 in MCF-7 cells. RT-qPCR analysis of T-UCRs and miR-221 levels. (**A**), transfection using miR-221 mimic molecule and evaluation of T-UCRs expression; (**B**), transfection using T-UCR siRNAs and evaluation of pre-miR-221 and miR-221. The relative expression was normalized on the mock transfection and calculated as 2^−∆∆Cq^. Values reported are the means of 4 experiments ± SEM. Statistical significance was determined by unpaired two tailed Student *t*-test. *p*-values < 0.01 (**), *p*-values < 0.001 (***).

For this reason, we further studied the possible relationship between T-UCRs and miR-221, in synchronized MCF-7 cells using another approach, i.e., evaluating the expression of T-UCRs upon transfection with synthetic miR-221. Indeed, uc.183, uc.110, and uc.84 decreased at very low levels after treatment with the miR-221 mimic molecule (Figure 3A).

Conversely, we also evaluated the levels of both pre-miR-221 and miR-221 following MCF-7 transfection with T-UCRs’ siRNAs. As shown in Figure 3B, miR-221 displayed increase levels after treatment with uc.183 and uc.96 siRNAs.

Summarizing the data obtained considering these T-UCRs, the uc.183 was the only effective in all the investigated systems and seems to be the best candidate to interfere with miR-221 expression in inverse manner and dependently of S phase of cell cycle. Other T-UCR, namely uc.84 and uc.110, were also modulated during the cell cycle and showed a negative response in vitro to miR-221 up-regulation. However, unlike uc.183, these two T-UCR could not reciprocate and appeared as simply downstream of miR-221.

### 3.3. Downstream Effectors of T-UCR Inhibition

**Figure 4:**
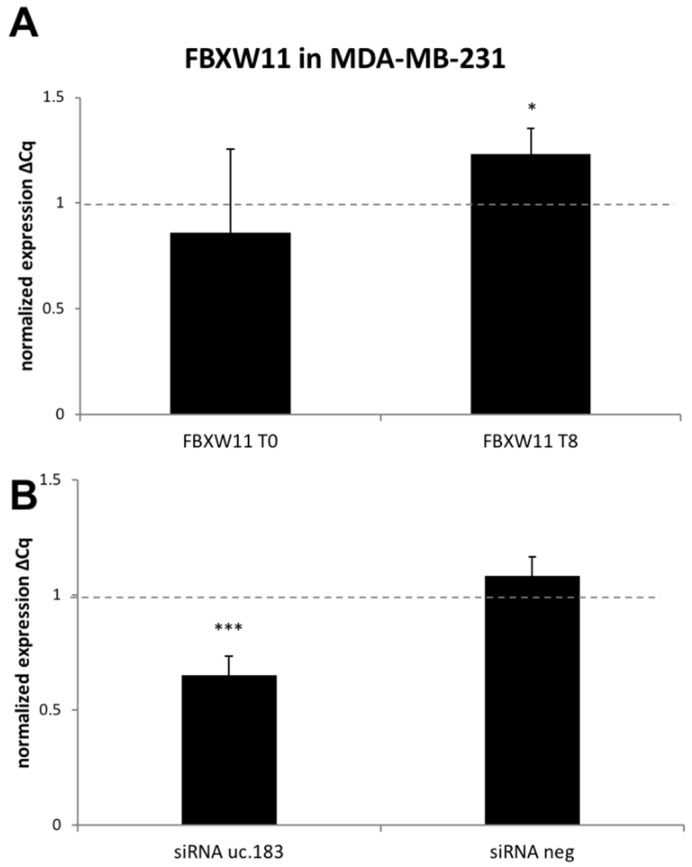
RT-qPCR analysis of FBXW11 mRNA in synchronized MDA-MB-231 cells. (**A**) *FBXW11* mRNA levels evaluated at T0 (release from double thymidine block) and T8 (8 h after release). (**B**) *FBXW11* mRNA levels analyzed in silenced cells with siRNA against uc.183 or siRNA negative control. The values were expressed as log2 fold changes quantified using 2^−ΔCq^ formula with respect to control. Statistical significance was determined by standard two-tailed Student *t*-test, *p*-value < 0.05 (*), *p*-value < 0.001 (***), derived from *n* = 4 independent replicates.

Since uc.183 is localized on a *FBXW11* coding exon (Table 2, Figure S1), we investigated *FBXW11* mRNA expression in synchronized MDA-MB-231 cells (either at T0 or T8), and any effect determined by T-UCR siRNAs. *FBXW11* levels were apparent at T8 (Figure 4A), thus siRNA treatment was performed in this cell culture condition.

As displayed in Figure 4B, transfection with siRNAs against uc.183 led to down-regulation of *FBXW11* expression at T8 suggesting an involvement also of the protein-coding gene in the network under miR-221/uc.183 control [49].

**Figure 5:**
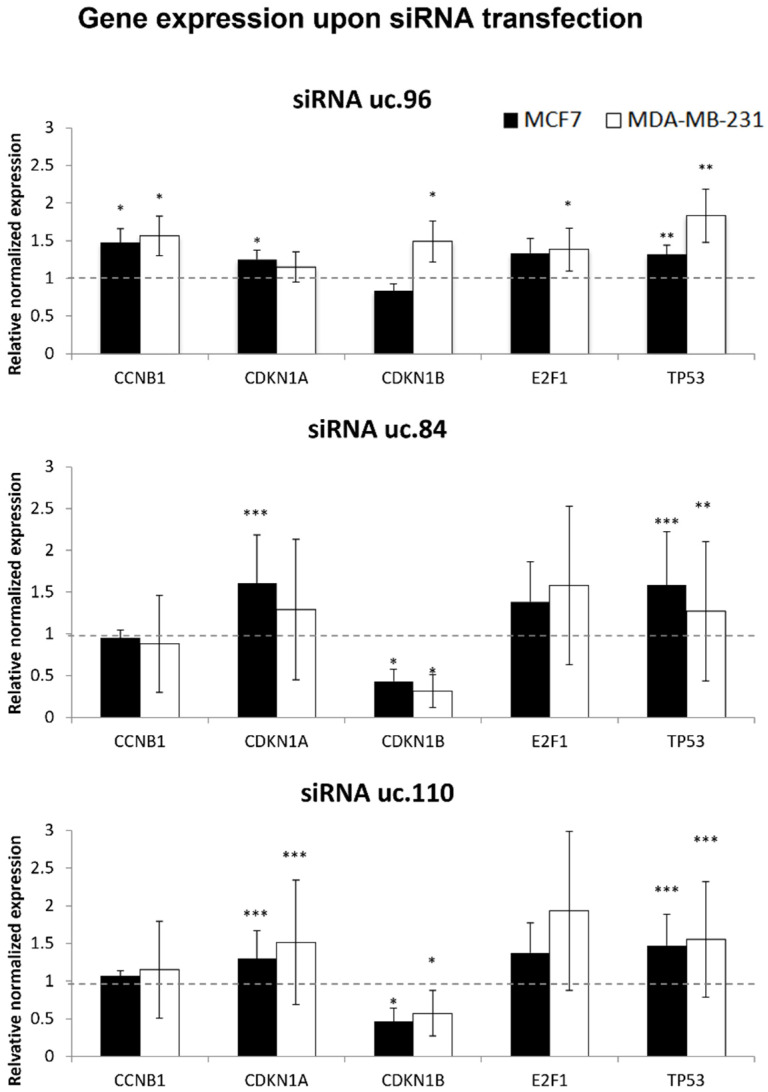
Quantitative analysis of miR-221 targets and cell cycle genes upon T-UCR siRNA transfection. Quantification by RT-qPCR demonstrated modulation of gene expression after treatment, calculated with respect to mock transfections. The dashed line parallel to the X axis indicates control relative expression of 1. Histograms represent the means of 8 independent experiments ± SEM. Statistical significance was determined by unpaired two tailed Student’s *t*-test; *p*-value < 0.05 (*), *p*-value < 0.01 (**), *p*-value < 0.001 (***).

Therefore, we enlarge our study investigating the effects of T-UCR perturbation, to include some genes known to be associated with the cell cycle and miR-221, e.g., *CDKN1B*, *TP53* and *E2F1* (known to be regulated by miR-221 [50,51,52]), as well as *CCNB1* and *CDKN1A* (Figure 5).

Analyzing the levels of these transcripts, we observed that uc.110, uc.96, and uc.84 siRNAs significantly up-regulated *TP53*, *E2F1*, and *CDK1A* in at least one cell line, while the uc.96 siRNA was effective on the rise of *CCNB1*. The effects of uc.110 and uc.84 were consistent with their interference in cell cycle; indeed, they caused also a strong down-regulation of *CDKN1B*, a known target of miR-221 [24].

### 3.4. Modulation of T-UCR Levels by Anticancer Drugs

**Figure 6:**
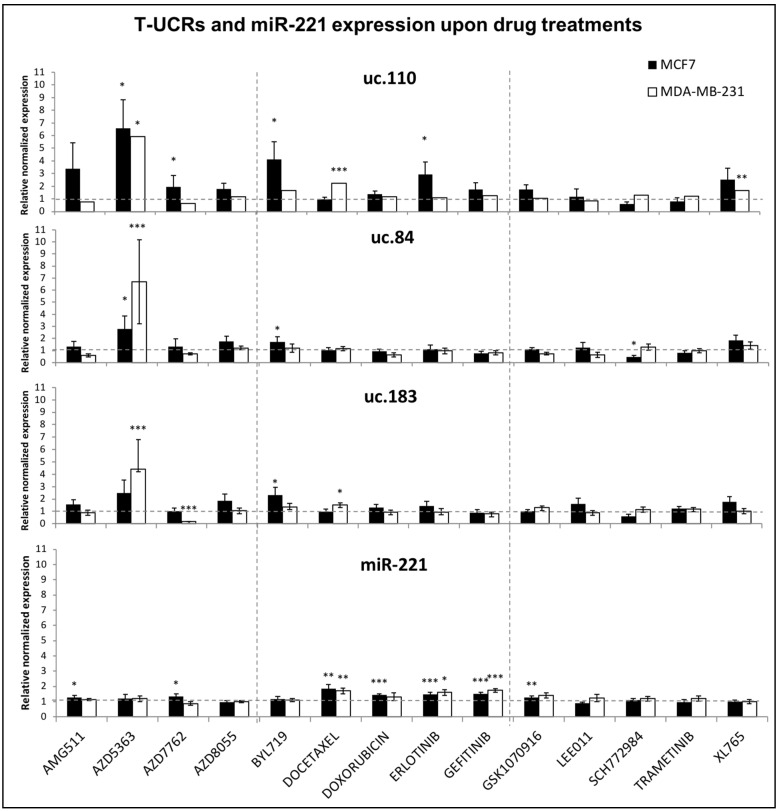
Gene expression analysis of T-UCRs and miR-221 upon treatment with anticancer drugs. Histograms describe the expression of uc.110, uc.84, and uc.183 detected by RT-qPCR and quantified by comparison with untreated cells using 2^−∆∆Cq^ formula. Values are mean of 5 experiments ± SEM. For statistical analysis, unpaired and two tailed Student’s *t* test has been used; adjusted *p*-values < 0.05 (*), *p*-value < 0.01 (**), *p*-value < 0.001 (***). Benjamini–Hochberg correction (FDR < 0.05) (Table S4). The dashed line parallel to the X axis indicates control relative expression of 1.

Since anticancer drugs often affect pathways related with the cell cycle, we investigated their possible action as modulators of T-UCRs. We used 14 drugs targeting the most frequently activated pathways in BC. We focused on the T-UCRs which were shown here to be experimentally involved in miR-221 activity or in the cell cycle. Therefore, we selected uc.183, which seemed to be entangled with miR-221 in a sort of negative loop, and uc.110 and uc.84 that seemed to succeed in the modulation of some cell cycle genes. We hypothesized a rise of these T-UCRs following the inhibitory activity of cancer drugs on cell cycle. Figure 6 shows an increase in expression of all tested T-UCRs (uc.183, uc.110, uc.84), in at least one cell line, upon treatment with the PI3K pathway inhibitors, AZD5363 (capivasertib) and BYL719 (alpelisib) that leave miR-221 completely unaffected (significant increase above 2-fold changes compared with untreated cells).

The *p*-values were adjusted according to Benjamini and Hochberg, for correction of multiple testing (FDR = 0.05) (Table S4).

Interestingly, the expression of miR-221 was upregulated by a range of other compounds, including doxorubicin and gefitinib, which instead did not up-regulate the T-UCRs. Thus, the treatments which affected the T-UCR expression did no alter the miR-221 levels and vice versa. Thus, with the small molecules inhibitors, we could show a completely differential response by miR-221 and T-UCRs, confirming the mutual exclusion detected in the initial data mining study.

In general, the accumulation of T-UCR occurred mostly in MCF7, excluding docetaxel and XL765, which acted selectively on uc.110 and uc.183 in MDA-MB-231. The Chk inhibitor, AZD7762 and ERK1/2 inhibitor, SCH772984 were the only compounds leading to high down-regulation of a T-UCR, respectively uc.183 in MDA-MB-231 and uc.84 in MCF7.

## 4. Conclusions

Notably, ncRNAs, such as T-UCRs are linked to cancer [53,54] via various mechanisms such as miRNA regulation [55]. In this context, miR-221 is one of the most relevant miRNAs in association with tumorigenesis [24], cell proliferation, invasion [25] malignancy, and metastasis [56]. In addition, miR-221 plays a pivotal role in cell cycle control [25] driving G1/S transition by targeting cyclin-dependent kinase inhibitors, p27 and p57 [24]. The aim of this work was to discover ncRNAs involved in the regulation of miR-221 and cell cycle. To identify candidate RNAs, we studied a very large dataset of tumors and normal RNA profiles, including data from over 1000 T-UCRs and miRNAs. Amongst them, 13 T-UCRs displayed inverse co-regulation with miR-221, e.g., were strongly expressed in the absence of miR-221 and vice versa. For the purposes of our research, we focused only on uc.183, uc.110, uc.96, and uc.84, the most effective in modulating cell cycle phases with their respective siRNA. Our observation on T-UCRs are novel, as there are no other reports in the literature, not only in breast cancer, but also for other cancer types.

We further investigated the relationship between these selected T-UCRs and miR-221, analyzing RNA interference of uc.84, uc.96, uc.110, and uc.183 on the cell cycle in synchronized BC cell lines. The results confirmed the mutually exclusive roles for miR-221 and the T-UCRs. In fact, the treatment with siRNAs against uc.183 and uc.96 increased cells in the S phase, just like miR-221 mimics. Additionally, miR-221 reduced the expression of uc.183, uc.110, and uc.84, and conversely, siRNAs against uc.183 and uc.96 increased pre-miR-221 and miR-221.

By investigating the role of T-UCRs in the control of cell cycle, we demonstrated that siRNAs against uc.110, uc.96, and uc.84 up-regulated *TP53*, *E2F1*, and *CDK1A*, whilst uc.110 and uc.84 siRNAs led to reduction of levels of *CDKN1B*, one of the most important targets for miR-221 [24]. Moreover, siRNA against uc.183 is associated with a downregulation of *FBXW11*. Lastly, the siRNAs against uc.96 solely up-regulated *CCKNB1*. Thus, T-UCRs appeared to be involved in the regulation of some key cell cycle genes, and, in particular, uc.110 and uc.84 to be engaged with CDKN1B.

We further dissected the miR-221 and T-UCR response in vitro, using a set of cancer drugs. The drugs targeting PI3K (AZD5363, AZD7762, AZD8055) and mTOR pathway (XL765) [57] determined an over-expression of T-UCRs that was predominant in MDA-MB-231 cells, while BYL719, which directly targets PIK3CA, was borderline effective only in MCF-7 cells, possibly because the mutations of PIK3CA (E542K and E545K) are not present in MDA-MB-231 cells [58].

We can conclude that T-UCRs sustain cell cycle modulation in two cell line models of breast cancer. Additionally, uc.183, uc.110, and uc.84 are mutually exclusive of miR-221, and seem to be components of alternative cell cycle circuits.

## Figures and Tables

**Table 1:** Data mining results for co-regulations of T-UCRs and miR-221. The name and genomic strand of both miRs (MATURE) and T-UCRs (ULTRACONS) correlated with miR-221 (bait) are reported, after selection of those with MAS ≥ 0.01, MIC ≥ 0.2, and abs(r) ≥ 0.4 threshold. The OSU microarray chip has probes for mature miRNAs (which tend to be conserved in the genomic sequences) and ultraconserved UCRs.

Bait	OSU Chip Definition	ncRNA	Genomic Strand	MIC (Strength)	MAS (Non Monotonicity)	Pearson Correlation (r)	Type of Correlation
miR-221	MATURE	hsa-miR-222	+	0.42	0.03	0.70	direct
miR-221	ULTRACONS	uc.84	−	0.32	0.03	−0.55	inverse
miR-221	MATURE	hsa-miR-634	+	0.28	0.01	−0.52	inverse
miR-221	ULTRACONS	uc.340	+	0.26	0.04	−0.49	inverse
miR-221	ULTRACONS	uc.478	−	0.26	0.01	−0.49	inverse
miR-221	ULTRACONS	uc.167	+	0.25	0.02	−0.50	inverse
miR-221	MATURE	hsa-miR-497	+	0.25	0.02	−0.43	inverse
miR-221	MATURE	hsa-miR-26b	+	0.24	0.04	0.43	direct
miR-221	MATURE	hsa-miR-26a	+	0.24	0.06	0.40	direct
miR-221	ULTRACONS	uc.110	−	0.24	0.04	−0.45	inverse
miR-221	MATURE	hsa-miR-602	+	0.24	0.04	−0.45	inverse
miR-221	ULTRACONS	uc.31	+	0.24	0.02	−0.43	inverse
miR-221	MATURE	hsa-miR-320	+	0.23	0.01	0.45	direct
miR-221	ULTRACONS	uc.10	−	0.23	0.01	−0.47	inverse
miR-221	ULTRACONS	uc.48	−	0.23	0.02	−0.48	inverse
miR-221	ULTRACONS	uc.78	+	0.23	0.01	−0.44	inverse
miR-221	MATURE	hsa-miR-361-5p	+	0.23	0.02	0.45	direct
miR-221	ULTRACONS	uc.183	+	0.22	0.04	−0.43	inverse
miR-221	ULTRACONS	uc.96	+	0.22	0.03	−0.41	inverse
miR-221	ULTRACONS	uc.309	−	0.21	0.01	−0.47	inverse
miR-221	MATURE	hsa-miR-30a	+	0.20	0.02	0.43	direct
miR-221	ULTRACONS	uc.177	−	0.20	0.01	−0.43	inverse

**Table 2:** Genomic coordinates and characteristics of T-UCRs, candidate alternatives/antagonists of miR-221.

T-UCR	Strand	Chromosome Coordinates (hg19)	Chromosome Coordinates (hg38)	Length (nt)	Type	Annotations
uc.84	−	chr2:157194706-157194914	chr2:156338194-156338402	209	exonic/ intronic	AK128708/intron of NR4A2; possible coding exon (42 amino acids starting with MET)—no known homology—Immediate-early response gene of the steroid-thyroid hormone-retinoid receptor superfamily [36]
uc.340	+	chr12:54090832-54091090	chr12:53697048-53697306	259	intergenic	partially overlaps with TCONS_00020432 lincRNA
uc.478	−	chrX:122599457-122599708	chrX:123465606-123465857	252	exonic	antisense of GRIA3
uc.167	+	chr5:88179624-88179824	chr5:88883807-88884007	201	intronic	antisense of MEF2C
uc.110	−	chr2:237071382-237071624	chr2:236162738-236162980	243	intergenic	enhancer and overlaps with the transmap of GBX2, an embryonal transcription factor [37]
uc.31	+	chr1:88928018-88928270	chr1:88462335-88462587	253	intergenic	BC045705 upstream of TCONS_00001016/TCONS_00001015
uc.10	−	chr1:10965574-10965848	chr1:10905517-10905791	275	intergenic	none
uc.48	−	chr2:20478333-20478630	chr2:20278572-20278869	298	exonic	overlaps with sense PUM2
uc.78	+	chr2:145188354-145188601	chr2:144430787-144431034	248	intronic	antisense of ZEB2
uc.183	+	chr5:171384520-171384755	chr5:171957516-171957751	236	exonic	antisense of FBXW11 [38,39,40,41]
uc.96	+	chr2:172820674-172820934	chr2:171964152-171964412	261	intronic	intron of HAT1—possible novel exon-homology to a non-human HAT [42,43,44,45]
uc.309	−	chr10:103267031-103267298	chr10:101507274-101507541	268	intronic	antisense of BTRC
uc.177	−	chr5:170417629-170417885	chr5:170990625-170990881	257	intronic	antisense of RANBP17

## Data Availability

Data are available online at the GEO repository (GSE70180) as reported by Galasso et al. [8]. The sequences for all used siRNAs and PCR primers are reported in the Supplementary Tables.

## Supplementary Materials

The following are available online at https://www.mdpi.com/article/10.3390/genes12121978/s1, Figure S1. The T-UCR siRNA pool composition. siRNA against T-UCRs from Table 2 grouped in pool as described; Figure S2. The effect of siRNA pool transfection on cell cycle phases. Flow cytometer analysis after pools transfection in MCF10A and MCF7 cell lines shows a significant cell cycle modification calculated by Student *T*-test. (*) *p*-value < 0.05, (**) *p*-value < 0.01; Figure S3: RNA interference of T-UCR from Table 2 on cell cycle in MCF7. Cell cycle was analyzed after transfections with siRNAs against T-UCR reported in Table2. Quantification was plotted as log2 ratio (median); Figure S4: Genome browser overview of uc.183 siRNA; Figure S5: Genome browser overview of uc.96 siRNA; Figure S6: Genome browser overview of uc.110 siRNA; Figure S7: Genome browser overview of uc.84 siRNA; Figure S8: Basal T-UCR expression levels normalized to β-Actin housekeeping gene. Values are median ± SEM of triplicate experiments; Figure S9: RNA interference of uc.84, uc.96, uc.110 and uc.183 on cell cycle in BC synchronized cell lines. Quantification was plotted as mean fold change ± SEM. Statistical significance was calculated, and the result compared to control random siRNA by 2-tailed Mann–Whitney test. *p*-values < 0.05 (*), *p*-values < 0.01 (**), *p*-values < 0.001 (***). Table S1: SiRNAs sequences against T-UCRs; Table S2: Primers used in qPCRs to amplified specific targets; Table S3: NcRNAs expression in synchronized cells. The ncRNA levels were indicated as fold changes (FC) determined by qPCRs (2^−∆∆Cq^) in synchronized cells with double thymidine block or serum starvation compared to no synchronized negative control. Data represent the fold change values T0 (at the end of the block, cells arrested in G0/G1 phase) and T8 (8 h from release); Table S4: Benjamini–Hochberg adjustment of T-UCRs and miR-221 expression results after drug treatments of BC cell lines. (FDR < 0.05).

## Abbreviations

ncRNA	non-coding RNA
miRNA	microRNA
T-UCR	transcribed ultra-conserved region
UCR	ultra-conserved region
lncRNA	long non-coding RNA
BC	breast cancer
