# Supplementary material for: UC.183, UC.110, and UC.84 Ultra-Conserved RNAs Are Mutually Exclusive with miR-221 and Are Engaged in the Cell Cycle Circuitry in Breast Cancer Cell Lines
Source: Genes (Basel). 2021 Dec 13;12(12):1978. doi: 10.3390/genes12121978 (PMC8701292; doi:10.3390/genes12121978)
Supplement: Supplementary file 1 [file genes-12-01978-s001.zip › Figure S1.pdf]

Pool 1	uc.96, uc.48, uc.31
Pool 2	uc.84, uc.177, uc.167, uc.78
Pool 3	uc.340, uc.10, uc.110
Pool 4	uc.183, uc.31, uc.309

Figure S1. The uc. siRNA pool composition. siRNA from table 2 grouped in pool as described.
