# Supplementary material for: UC.183, UC.110, and UC.84 Ultra-Conserved RNAs Are Mutually Exclusive with miR-221 and Are Engaged in the Cell Cycle Circuitry in Breast Cancer Cell Lines
Source: Genes (Basel). 2021 Dec 13;12(12):1978. doi: 10.3390/genes12121978 (PMC8701292; doi:10.3390/genes12121978)
Supplement: Supplementary file 1 [file genes-12-01978-s001.zip › Figure S2.pdf]

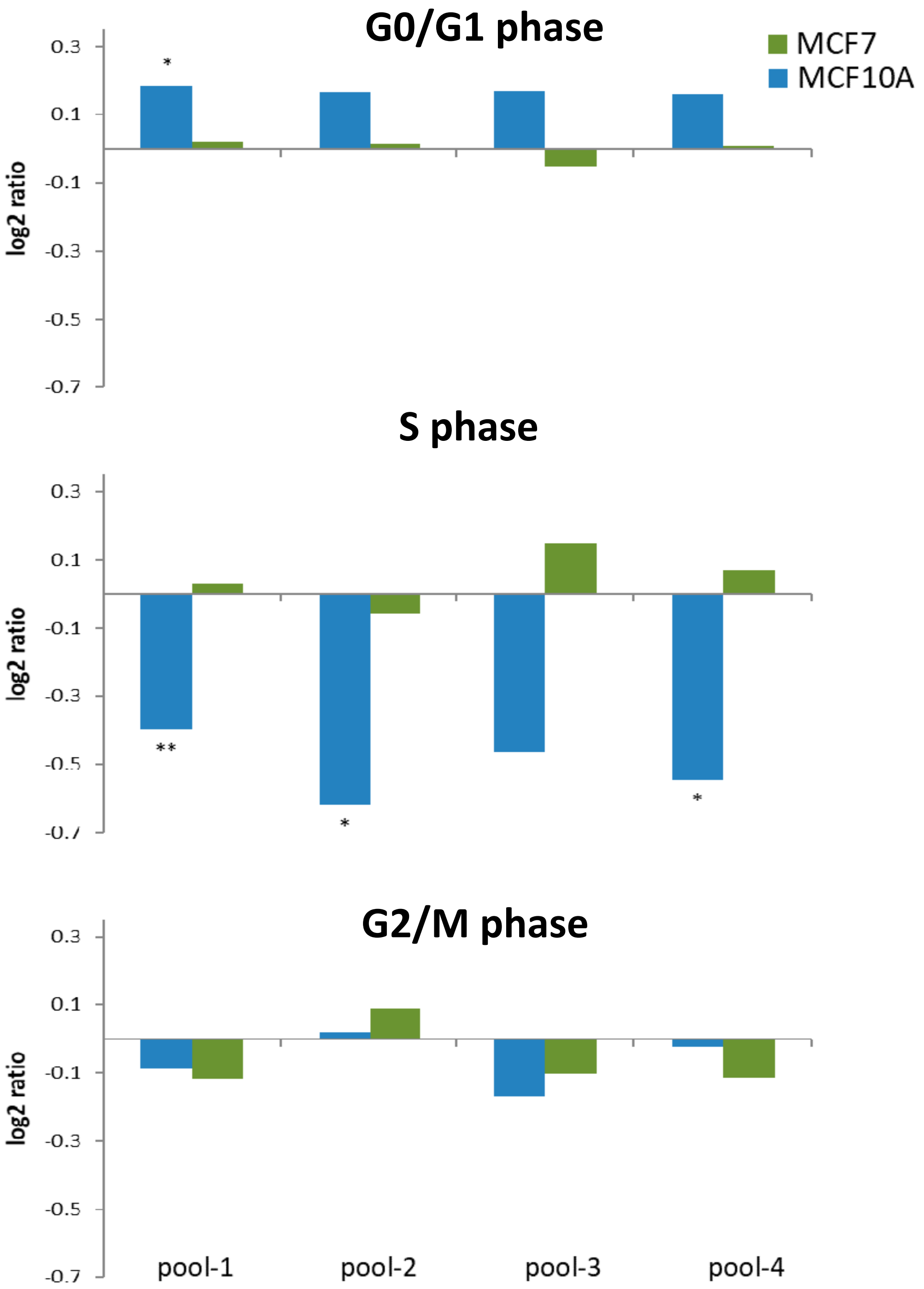

Figure S2. siRNA pool transfection effect in cell cycle. Flow cytometer analysis after pools transfection in MCF10A and MCF7 cell lines shows a significant cell cycle modification calculated by Student T-test. (*) p-value<0.05, (**) p-value <0.01.
