# Supplementary material for: UC.183, UC.110, and UC.84 Ultra-Conserved RNAs Are Mutually Exclusive with miR-221 and Are Engaged in the Cell Cycle Circuitry in Breast Cancer Cell Lines
Source: Genes (Basel). 2021 Dec 13;12(12):1978. doi: 10.3390/genes12121978 (PMC8701292; doi:10.3390/genes12121978)
Supplement: Supplementary file 1 [file genes-12-01978-s001.zip › Figure S3.pdf]

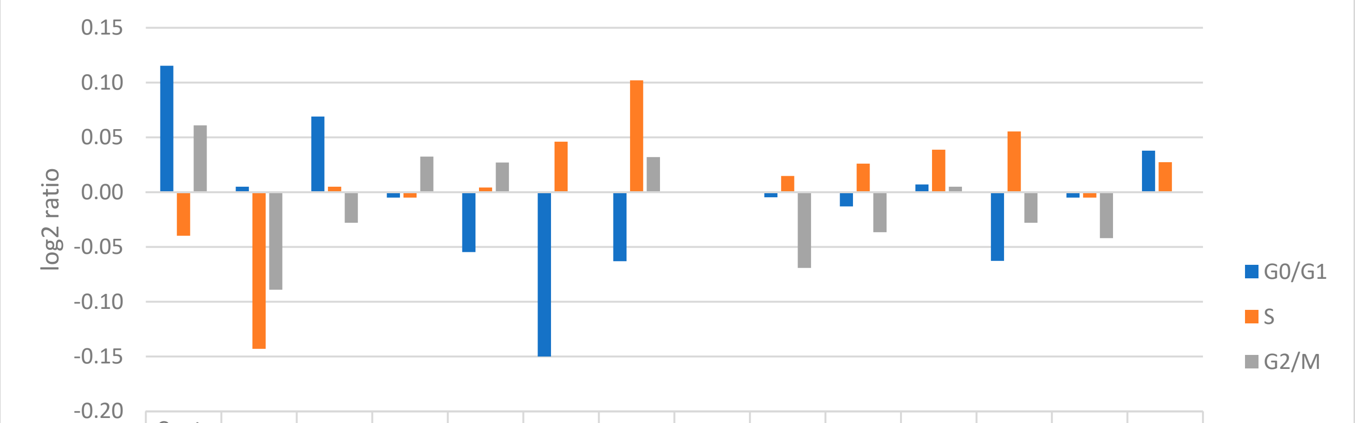

	Control siRNA	uc.96 siRNA	uc.48 siRNA	uc.31 siRNA	uc.177 siRNA	uc.84 siRNA	uc.167 siRNA	uc.78 siRNA	uc.340 siRNA	uc.10 siRNA	uc.110 siRNA	uc.183 siRNA	uc.31 siRNA	uc.309 siRNA
G0/G1	0.115	0.005	0.069	-0.005	-0.055	-0.150	-0.063	0.000	-0.005	-0.013	0.007	-0.063	-0.005	0.038
S	-0.040	-0.143	0.005	-0.005	0.004	0.046	0.102	0.000	0.015	0.026	0.039	0.055	-0.005	0.027
G2/M	0.061	-0.089	-0.028	0.032	0.027	0.000	0.032	0.000	-0.069	-0.037	0.005	-0.028	-0.042	

Figure S3. RNA interference of T-UCR from Table 2 on cell cycle in MCF7. Cell cycle was analyzed after transfections with siRNAs against T-UCR reported in Table2. Quantification was plotted as log2 ratio (median).
