# Supplementary material for: UC.183, UC.110, and UC.84 Ultra-Conserved RNAs Are Mutually Exclusive with miR-221 and Are Engaged in the Cell Cycle Circuitry in Breast Cancer Cell Lines
Source: Genes (Basel). 2021 Dec 13;12(12):1978. doi: 10.3390/genes12121978 (PMC8701292; doi:10.3390/genes12121978)
Supplement: Supplementary file 1 [file genes-12-01978-s001.zip › Figures S4-S7.pdf]

Supplementary information

Genomic annotation of the T-UCRs negatively associated with miR-221.

Since UCRs have been described over a decade ago, we carefully re-assessed the genomic annotation of each of the T-UCRs that we have selected on the basis of negative correlation with miR-221. Hereafter, we list some of the relevant details revealed in the re-assessment.

The genomic location of uc.183 is in the FBXW11 gene, in exon 2, but it is expressed in antisense orientation (Figure S4). FBXW11 is a member of the E3 ubiquitin ligase family and is regulated during tumorigenesis; however, its role remains ambiguous [38, 39]. Indeed, it has been reported to act both as tumor suppressor and oncogene [39]. In addition, FBXW11 is a member of the F-box protein family, also implicated in cancer development [41], which regulates checkpoints of cell cycle [40].

Regarding uc.96, this T-UCR is transcribed in sense strand from an intron of HAT1, (Figure S5), encoding a histone acetyltransferase which catalyzes the acetylation of histone H4 during replication-dependent chromatin assembly [42]. HAT-1 is up-regulated in cancer with poor prognosis [43] and is crucial for S-phase progression [44]; knockdown of this gene caused cell cycle arrest in G2/M[45]. HAT1 is also not a predicted target by miR-221/222, but it is a predicted target of the known ‘tumor suppressors’ miR-203 and miR-143 [2].

The other uc.110 T-UCR mapped within an intergenic region listed as enhancer sequence overlapping with the transmap of GBX2 [36] (Figure S6).

Finally, the uc.84 is located within AK128708 genomic region contained in the intronic region of NR4A2, an orphan nuclear receptor with many roles in BC such as breast transformation, cancer cell proliferation, and patient survival [35] (Figure S7).

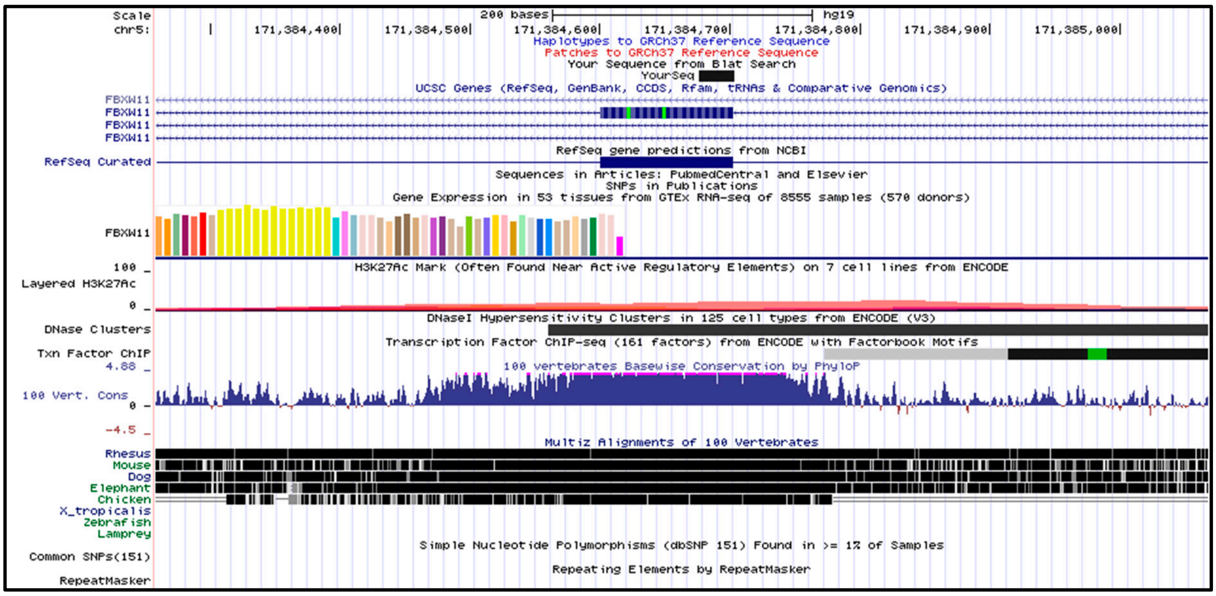

Figure S4. Genome browser overview of uc.183 siRNA (indicated as YourSeq).

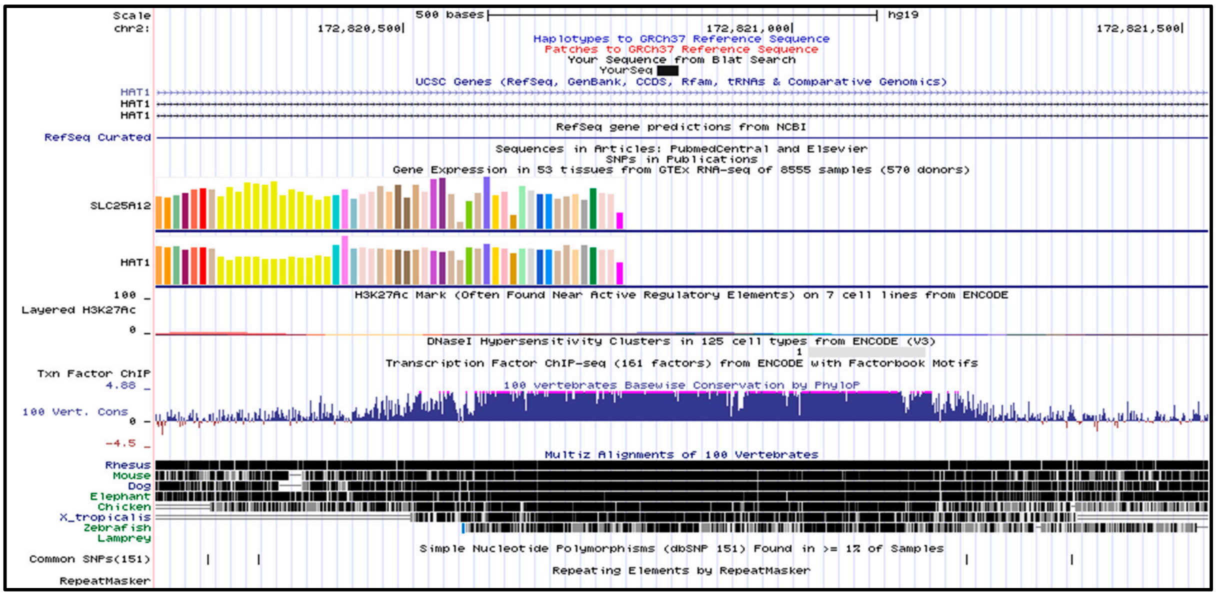

Figure S5. Genome browser overview of uc.96 siRNA (indicated as YourSeq).

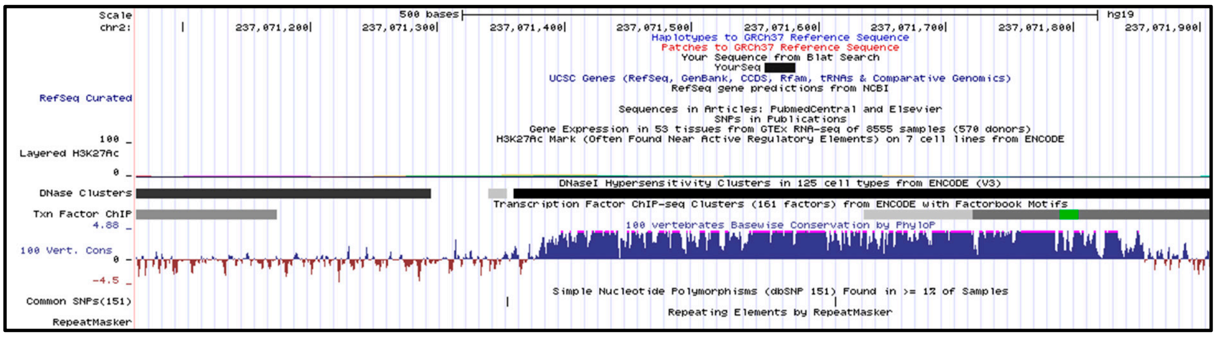

Figure S6. Genome browser overview of uc.110SS siRNA (indicated as YourSeq).

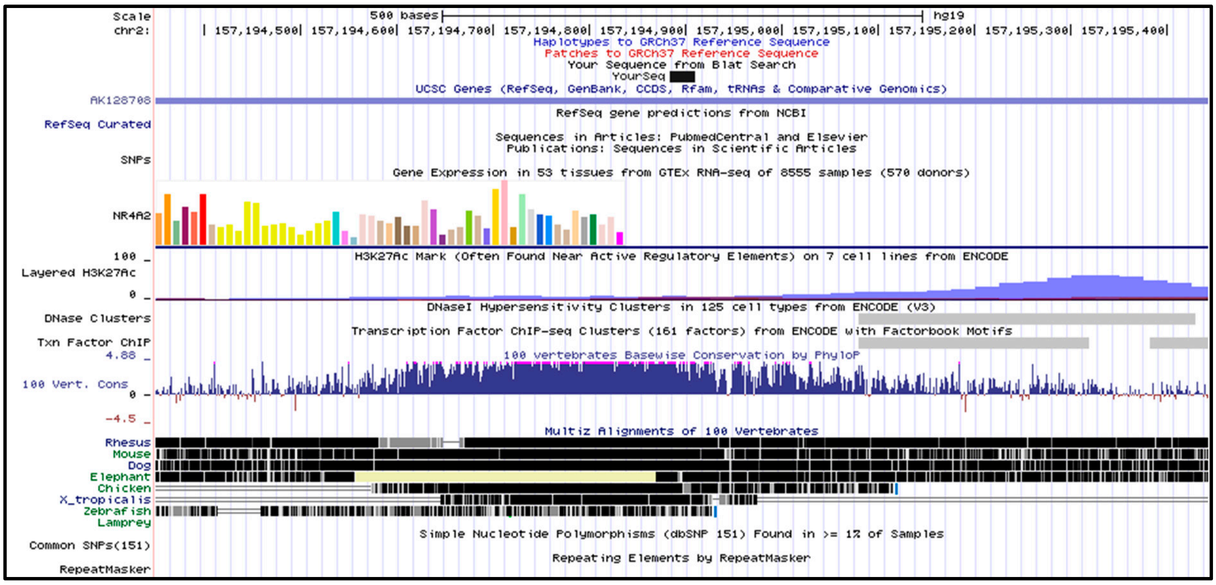

Figure S7. Genome browser overview of uc.84 siRNA (indicated as YourSeq).
