# Supplementary material for: UC.183, UC.110, and UC.84 Ultra-Conserved RNAs Are Mutually Exclusive with miR-221 and Are Engaged in the Cell Cycle Circuitry in Breast Cancer Cell Lines
Source: Genes (Basel). 2021 Dec 13;12(12):1978. doi: 10.3390/genes12121978 (PMC8701292; doi:10.3390/genes12121978)
Supplement: Supplementary file 1 [file genes-12-01978-s001.zip › Table S1.pdf]

Table S1. T-UCR siRNAs oligonucleotide sequences.

siRNA name	Sequence (5’→3’)	Genomic siRNA strand	Genomic strand T-UCR
uc.84	GGGAGGGAGCUAAUCAAGUUACAUCAA	+	-
uc.84	GAUGUAACUUGAUUAGCUCCCUCCC	-	-
uc.110	AUCAUUGGGAGUUAAUUUGAAGCUCAG	+	-
uc.110	GAGCUUCAAAUUAACUCCCAAUGAU	-	-
uc.183	GUGUUCUGUGCCAAGGUCUUUGUGGCU	-	+
uc.183	CCACAAAGACCUUGGCACAGAACAC	+	+
uc.96	AAGGACUGACACAAGAACACUUUGUGU	-	+
uc.96	ACAAAGUGUUCUUGUGUCAGUCCTT	+	+
