# Supplementary material for: UC.183, UC.110, and UC.84 Ultra-Conserved RNAs Are Mutually Exclusive with miR-221 and Are Engaged in the Cell Cycle Circuitry in Breast Cancer Cell Lines
Source: Genes (Basel). 2021 Dec 13;12(12):1978. doi: 10.3390/genes12121978 (PMC8701292; doi:10.3390/genes12121978)
Supplement: Supplementary file 1 [file genes-12-01978-s001.zip › Table S2.pdf]

Table S2. Primers used in qPCRs to amplified specific targets.

Name	Forward primer	Reverse primer
uc.84	CTGCTTGATGTAACTTGATTAGCTC	AAATCTACATCTATCATTTTGCCAAT
uc.96	ACCCATCATGCATGTAAACG	AATGGAGTTTGCCGTGAGTT
uc.110	CCGTCCTCCCTGCACTAA	CAGCCAAAGGGGAAGTGTAT
uc.183	CTGCCCTATCAACTTTCGATGGTAG	CCGTTTCTCAGGCTCCCTCTC
pre-miR-221	GAGAACATGTTTCCAGGTAG	CAGGTCTGGGGCATGAAC
CCNB1	AGGCGAAGATCAACATGGCA	GGGCTTGGAGAGGCAGTATC
E2F1	CTTGGAGGGACCAGGGTTTC	GTTCACCTTCATTCCCCGGT
TP53	TCACTTCCTTTTTCCGCAGT	TTCTGGCACTTCATCACAGC
CDKN1A	GCTTCATGCCAGCTACTTCC	CTGTGCTCACTTCAGGGTCA
CDKN1B	GTGCGAGTGTCTAACGGGAG	AGCCGGAGCCCCAATTAAAG
FBXW11	CAGCTAGAGTGGGTGCGAT	GGCCTCTTTCTGGAGACGAT
B-Act	CATTGCCGACAGGATGCA	GCTGATCCACATCTGCTGGA
