# Supplementary material for: UC.183, UC.110, and UC.84 Ultra-Conserved RNAs Are Mutually Exclusive with miR-221 and Are Engaged in the Cell Cycle Circuitry in Breast Cancer Cell Lines
Source: Genes (Basel). 2021 Dec 13;12(12):1978. doi: 10.3390/genes12121978 (PMC8701292; doi:10.3390/genes12121978)
Supplement: Supplementary file 1 [file genes-12-01978-s001.zip › Table S3.pdf]

Table S3. NcRNAs expression in synchronized cells. The ncRNA levels were indicated as fold changes (FC) determined by qPCRs ($2^{-\Delta\Delta Cq}$) in synchronized cells with double thymidine block or serum starvation compared to no synchronized negative control. Data represent the fold change values T0 (at the end of the block, cells arrested in G0/G1 phase) and T8 (8 h from release).

MCF-7					MDA-MB-231				
	**Condition**	**T0 (FC)**	**T8 (FC)**	**T8/T0 (FC)**		**Condition**	**T0 (FC)**	**T8 (FC)**	**T8/T0 (FC)**
pre-mir-221	Double thymidine block	0.36	2.56	7.09	pre-mir-221	Double thymidine block	0.52	2.21	4.21
	Serum starvation	24.01	12.25	0.51		Serum starvation	0.05	0.31	6.36
mir-221	Double thymidine block	0.31	4.56	14.66	mir-221	Double thymidine block	0.29	0.00	0.01
	Serum starvation	2.02	5.65	2.81		Serum starvation	9.98	0.01	0.00
uc.183	Double thymidine block	1.26	0.08	0.07	uc.183	Double thymidine block	12.27	0.14	0.01
	Serum starvation	3.71	0.01	<0.00		Serum starvation	6.86	0.15	0.02
uc.110	Double thymidine block	0.80	0.47	0.49	uc.110	Double thymidine block	4.75	0.05	0.01
	Serum starvation	0.15	0.15	1.00		Serum starvation	2.16	2.16	1.00
uc.96	Double thymidine block	3.82	0.67	0.18	uc.96	Double thymidine block	1.77	1.30	0.73
	Serum starvation	11.91	0.11	0.01		Serum starvation	17.43	0.82	0.05
uc.84	Double thymidine block	5.89	4.71	0.81	uc.84	Double thymidine block	0.01	0.16	16.08
	Serum starvation	3.79	5.50	1.45		Serum starvation	0.02	1.12	45.14
