# Supplementary figures and images for: UC.183, UC.110, and UC.84 Ultra-Conserved RNAs Are Mutually Exclusive with miR-221 and Are Engaged in the Cell Cycle Circuitry in Breast Cancer Cell Lines
Source: Genes (Basel). 2021 Dec 13;12(12):1978. doi: 10.3390/genes12121978 (PMC8701292; doi:10.3390/genes12121978)

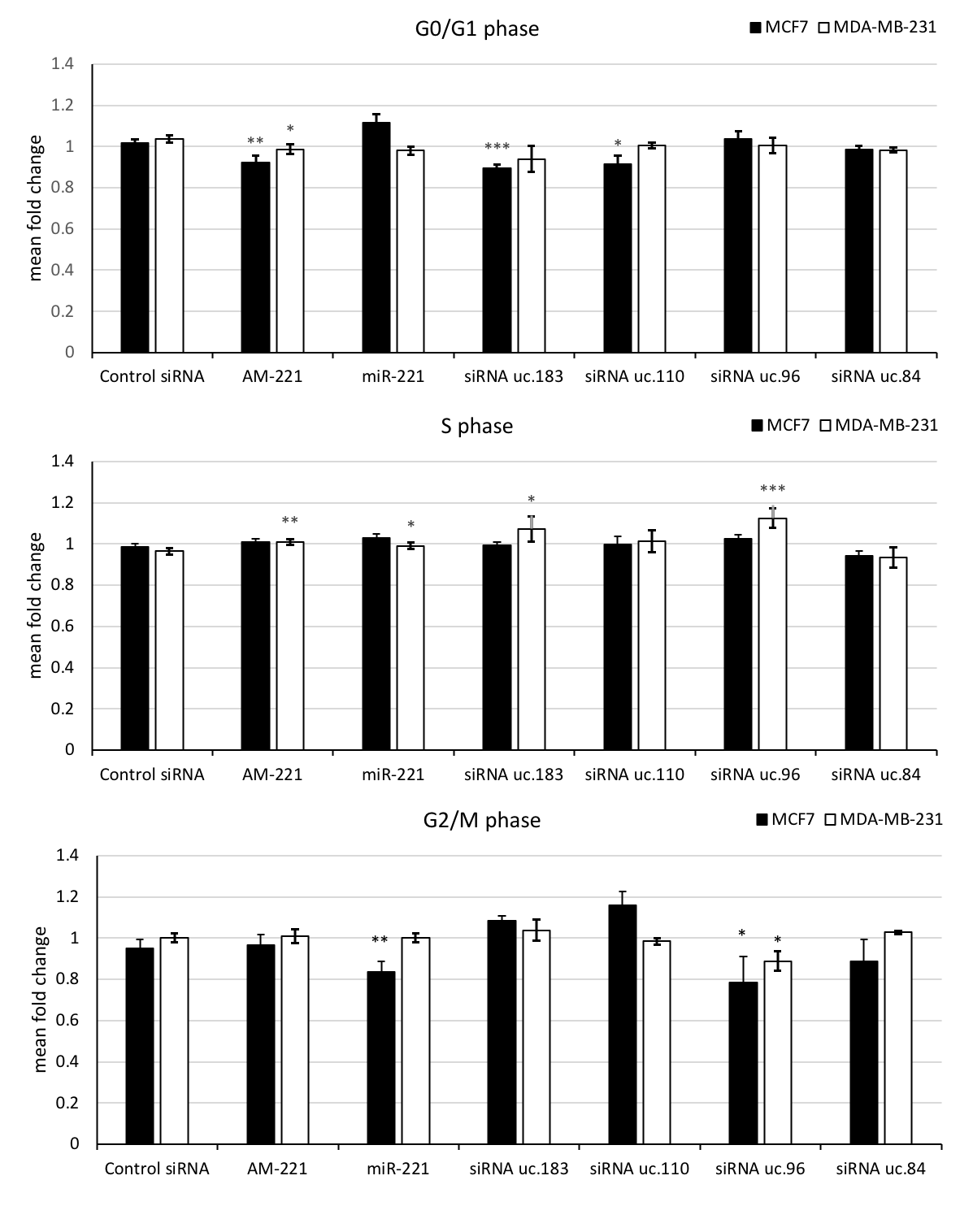

Supplement: Supplementary file 1 [file genes-12-01978-s001.zip › Figure S9.png]
